# Nanostructured and Functional Nanomaterials for Energy Storage and Removal of Pollutants

**DOI:** 10.3390/nano13192631

**Published:** 2023-09-24

**Authors:** Glaydson Simões dos Reis, Chandrasekar M. Subramaniyam

**Affiliations:** 1 Department of Forest Biomaterials and Technology, Biomass Technology Centre, Swedish University of Agricultural Sciences, SE-901 83 Umeå, Sweden; 2 Department of Chemistry and Biochemistry, Faculty of Pharmacy, Universidad San Pablo-CEU, CEU Universities, Boadilla del Monte, 28668 Madrid, Spain; mayandi@ceu.es

Nanomaterials have a long history, and people have utilized them unknowingly. Nanomaterials can be characterized as particles existing in nature or artificially synthetized materials with one or more external dimensions in the 1–100 nm range and are mostly made up and or composed of carbon, silicon, metal and their oxides, etc. [1]. The challenge for researchers working in the field of nanomaterials is the ability to work at these levels to generate larger structures with fundamentally new atomic, molecular, or particle organization [1]. These structures can be suitable for a wide range of applications according to their molecular, structural and surface properties [1,2]. Nanostructured materials may possess important properties such as a high specific surface area and a large number of functional groups on their surfaces, and new mechanical, electronic, magnetic, and adsorptive properties, as well as catalytic activity. All of these properties have great potential to revolutionize several fields, including energy storage and water decontamination [2,3]. The prospects for their application are very diverse, mainly due to their different physicochemical characteristics, making them very suitable for the aforementioned applications (not limited) in areas such as energy storage (electrodes for supercapacitors and batteries) and environmental applications (water decontamination).

Nanostructured material properties can be easily modified/tailored as desired via different synthesis methods, which precisely control their size, shape, synthesis conditions, and appropriate surface functionalization. Two main synthesis approaches are employed for the preparation of nanostructured materials [4]. One so-called top-down approach consists of various methodologies, including mechanical milling, electrospinning, sputtering, laser ablation methods, etc. [4]. The second approach (bottom-up) includes chemical vapor deposition, thermochemical methods, sol–gel, etc. [4]. It is worthwhile to highlight that the characteristics of these nanostructured materials are severely dependent on the synthesis methods used to manufacture them; therefore, full knowledge of them is very important in order to obtain materials with desired properties for suitable/specific applications.

The present Special Issue contains eight papers [5,6,7,8,9,10,11,12] devoted to the synthesis and application of nanostructured materials for water decontamination through adsorption, photocatalysis and filtration and the building of high energy density energy storage devices. The papers present different nanostructured materials such as Doped biomass-activated carbon materials, Titanate Perovskite-Based Nanocomposites, composites based on Graphene Oxide/TiO_2_, Nano-Clay, Three–Dimensional Na_3_V_2_(PO_4_)_3_/Carbon Frameworks, and Nanostructured ZnO.

These nanomaterials have been applied in the decontamination of polluted synthetic waters, including the adsorption of dyes [6,7], the filtration of heavy metals [6], adsorption coupled with ultrafiltration to remove nitrite ions [8], and the removal of dyes and organic contaminants using photocatalysis [9,10] to the degradation of dyes and pharmaceuticals, along with heavy metal ion and radioactive ion extraction.

Regarding the employment of these nanostructured materials for energy storage applications, this Special Issue contains two contributions on the synthesis and application of three-dimensional (3D) Na_3_V_2_(PO_4_)_3_/holey-carbon frameworks as cathodes for sodium-ion batteries [11] and a review paper on biomass-derived carbon materials for potassium and aluminum batteries [12].

The construction of next-generation technological processes is impossible without using an improvised material base, so nanostructured and functional materials are of greater importance to go beyond our societal needs. The prospects for their application are very diverse, mainly due to their different and adaptable physicochemical characteristics, which are pretty much needed in all technological areas, including energy storage devices and environmental applications (water decontamination), but not only limited to these.

Overall, this Special Issue may contribute to the field of interest concerning the green synthesis of nanostructured materials for environmental and energy storage applications, providing our readership with relevant information on some of the latest prospects in these fast-evolving and cross-disciplinary areas.
